# First Detection and Identification of FAdV-8b as the Causative Agent of an Outbreak of Inclusion Body Hepatitis in a Commercial Broiler Farm in Greece

**DOI:** 10.3390/vetsci9040160

**Published:** 2022-03-25

**Authors:** Vasileios Tsiouris, Tilemachos Mantzios, Konstantinos Kiskinis, Jean-Luc Guérin, Guillaume Croville, Georgia D. Brellou, Emmanouela P. Apostolopoulou, Evanthia J. Petridou, Ioanna Georgopoulou

**Affiliations:** 1 Unit of Avian Medicine, Clinic of Farm Animals, School of Veterinary Medicine, Aristotle University of Thessaloniki, 54627 Thessaloniki, Greece; biltsiou@vet.auth.gr (V.T.); kiskinik@vet.auth.gr (K.K.); ioannag@vet.auth.gr (I.G.); 2 IHAP, Université de Toulouse, INRAE, ENVT, 31300 Toulouse, France; jean-luc.guerin@envt.fr (J.-L.G.); guillaume.croville@envt.fr (G.C.); 3 Laboratory of Pathology, School of Veterinary Medicine, Aristotle University of Thessaloniki, 54627 Thessaloniki, Greece; mprellou@vet.auth.gr (G.D.B.); emmaapos@vet.auth.gr (E.P.A.); 4 Laboratory of Microbiology and Infectious Diseases, School of Veterinary Medicine, Aristotle University of Thessaloniki, 54124 Thessaloniki, Greece; epetri@vet.auth.gr

**Keywords:** Inclusion Body Hepatitis (IBH), Fowl Aviadenoviruses (FAdVs), liver, pancreas, histopathology, phylogenetic analysis

## Abstract

Inclusion body hepatitis (IBH) is an economically important disease of chickens, with a worldwide distribution, caused by Fowl Aviadenoviruses (FAdVs). Currently, the increased number of cases, the virulence of the isolate strains, as well as the lack of cross-species protection highlight that detailed in-field data are fundamental for the development of successful control strategies. This case report provides a detailed clinicopathological investigation of an unusual IBH outbreak in a commercial broiler farm in the region of Macedonia, Greece. The farm consisted of 64,000 birds, originated from the same breeder stock and placed in three different houses (Flock A–C). At 20 days of age, a sudden increase in daily mortality was recorded in Flock A. It is worth mentioning that, although all flocks were serologically (indirect ELISA) and molecularly (RT-PCR) positive for FAdV, the mortality rate, attributed to IBH, was much higher in Flock A compared to others. The clinical manifestation included non-specific symptoms such as depression, inappetence, yellowish mucoid diarrhea, and lack of uniformity. At necropsy, typically, enlarged, pale, and friable livers were dominant, while sporadically lesions were recorded in the pancreas, kidneys, skeletal muscles, and lymphoid organs. The histopathological examination of liver samples showed multifocal inflammation, necrosis, and the presence of basophilic/ eosinophilic inclusion bodies in hepatocytes. In addition, the loss of the architecture of pancreatic lobules and the presence of fibrosis and foci of mononuclear cell aggregates were suggestive of chronic pancreatic inflammation. PCR analysis confirmed the presence of FAdV, belonging to species E, serotype FAdV-8b. Performance and financial calculations revealed that IBH increased Feed Conversion Ratio (FCR), feed cost/chick as well as feed cost/kg live weight, whereas the Livability (%) and the European Production Efficiency Factor (EPEF) were decreased in the most severely affected flocks (Flock A). This study is the first report of the detection and identification of FAdV serotypes associated with IBH in commercial broiler flocks in Greece. However, there is still a lack of information about the circulating FAdV serotypes in the country, and therefore epidemiological studies are needed to establish control strategies for IBH.

## 1. Introduction

Inclusion Body Hepatitis (IBH) is a viral disease of poultry caused by Fowl Aviadenoviruses (FAdVs) [1]. The name of the disease is attributed to the presence of characteristic basophilic and/or eosinophilic inclusion bodies, which are frequently observed in the affected hepatocytes, and are enough for histopathological diagnosis [2]. Since its first report in the early 60s, in the USA [3], the disease has spread worldwide [1,4,5,6]. In many areas, IBH became endemic, threatening the local poultry industry by subjecting afflicted flocks to mortality, growth retardation, and low productivity in general [4,5,7]. Long-term epidemiological surveys reveal that broilers of 3–5 weeks of age are more susceptible to the disease, whereas layer and breeder flocks of varying ages can also be affected [1,8,9].

Molecular investigation of the in-field isolates allows the differentiation of FAdVs to species and serotypes and provides a useful tool for epidemiological surveys and for the development of control programs in the affected regions [10]. FAdVs are molecularly divided into five species (FAdVA-E) and further classified into 12 serotypes (FAdV1-8a and FAdV8b-11) based on cross-neutralization tests [11]. The majority of FAdV serotypes (FAdV-2, 8a, 8b, and 11) are isolated from cases of IBH [1,12,13]. Nevertheless, certain serotypes are associated with other noteworthy poultry pathologies, including Hydropericardium Syndrome (FAdV-4) [14] and Gizzard erosion and Ulceration syndrome (FAdV-1) [15,16]. Finally, the same serotypes are frequently isolated from healthy birds, a fact which historically led to a complacency approach of FAdVs as primary pathogens [2].

The full mechanism of FAdVs to induce IBH has not been elucidated, and despite the successful reproduction of the disease in SPF flocks [9,17], there is a wide variation among experimental and in-field data, supporting that other factors are required for FAdVs to induce IBH in the farm. In epidemiological surveys from Mississippi, India, Egypt, Korea, and Belgium, outbreaks of IBH have been linked with immunosuppressive agents, including Infectious Bursal Disease Virus (IBDV) [5,7] Anemia Virus (CAV) [5,18], mycotoxins [19], and coccidiosis [20]. However, in regions such as Canada, Iran, Japan, and Australia, FAdVs were identified as primary pathogens to induce IBH in the field, without the presence of predisposing factors [21,22,23,24]. Therefore, in-field data are fundamental information to monitor and establish better control strategies at a national level, including management and successful vaccination programs [6].

Among European countries, FAdV 2, 8a,-8b, and 11 were found as the causative agent of IBH cases in broiler and breeder flocks in Spain during 2011–2013 [9]. IBH cases have also been reported in Slovenia [25], Poland [26,27,28], Hungary [25], Greece [29], Belgium [20], Germany, France, and Austria [12], with the majority of FAdV isolates belonging to species D and E, following the global pattern [1,6].

In Greece, since 2007, sporadic outbreaks of IBH in broilers were diagnosed based on necropsy and histopathological criteria [30]. In 2016, the presence of FAdVs was firstly recorded, based on the hexon loop-1 region and fiber gene, in IBH cases of 27-day-old broiler (LN907537, LN907567) and 18-day-old breeder flocks (LN907537, LN907563) [12]. The causative agents of these outbreaks were identified as genotype E3 and associated with 15% and 8.2% mortality in broiler and breeder flocks, respectively. Thereafter, in outbreaks of IBH during 2017–2018 in the geographical region of Epirus, circulating serotypes were found to belong mainly to FAdV-E (85.29%) and FADV-D (14.71%) [29]. It is important to note that commercial flocks in Greece are not subjected to vaccination and therefore are vulnerable to horizontal transmission.

There is scarce data available about the IBH serotype circulation in Greece, whereas the association of IBH with stress factors has never been investigated in the Greek poultry industry. Thus, the aim of this study was (1) to provide phylogenetic data about the isolated strain associated with an IBH outbreak that happened in the Regional Unit of Macedonia, Greece; (2) to record the clinical manifestation of the disease and determine the actual cost in the productivity of a Greek commercial broiler farm; and (3) to find out whether this outbreak was associated with IBDV, CAV, or the presence of mycotoxins in the feed.

## 2. Materials and Methods

### 2.1. Case History

#### 2.1.1. Flock History

On May of 2018 an IBH outbreak was recorded in a commercial broiler farm, which was placed in the region of Macedonia Greece and consisted of three poultry houses, placed in parallel formation, Flock A (21,800 birds) // Flock B (21,800 birds) // Flock C (20,400 birds), and summed a total of 64,000 birds that originated from the same breeder stock, were hatched at the same hatchery, and placed on the same date. The birds were vaccinated against Newcastle disease (ND) (AVINEW^®^, Boehringer Ingelheim Group, Ingelheim am Rhein, Germany) and Infectious Bronchitis (IB) (Cevac IBird, Ceva Animal Health Ltd., Libourne, France) by spray vaccination as well as against Infectious Bursal Disease (IBD) (CEVAC^®^ TRANSMUNE IBD, Ceva Animal Health Ltd., Libourne, France) by subcutaneous vaccination on the 1st day in the hatchery. Water and feed were offered to all birds *ad libitum*, whereas lighting program and microenvironmental conditions were automatically regulated to all houses according to the recommendations of the breeding company [31].

#### 2.1.2. Clinical Manifestation

**Figure 1:**
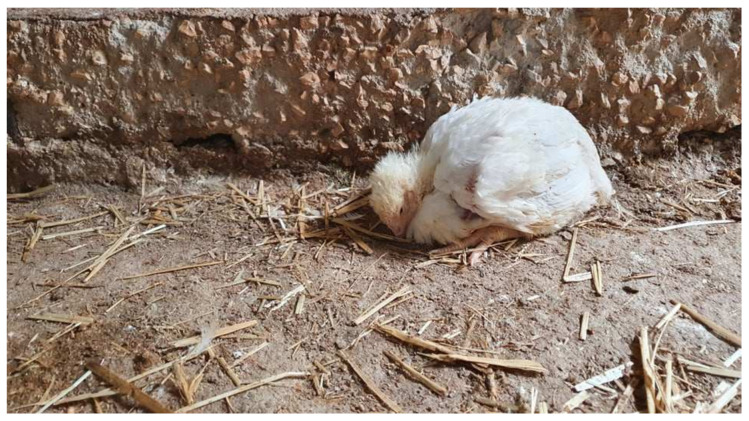
An affected 23-day old broiler chicken from Flock A. The bird revealed non-specific clinical signs including depression, lethargy, ruffling feathers, and inappetence.

At 20 days of age, a sudden onset in daily mortality alongside with nonspecific clinical signs was recorded in Flock A. In particular, the farmer’s concern arose from the fact that daily mortality increased exponentially with dead birds in Flock A, numbered approximately 250 at day 20 to 600 at day 26, whereas a rising proportion of clinical signs and mortality was recorded also in Flock B. The birds showed apathy, maintained crouching position with ruffled feathers (Figure 1), or huddled together. Autopsies of dead birds were suggestive for IBH, and thus strict biosecurity measures were immediately applied in the farm, while a commercial poultry hepatoprotectant (HEPATO PROTECT, Farmavet S.A., Bucharest, Romania) blend of silymarin, amino-acids, vitamins, and electrolytes was supplied to birds through drinking water (1lt/tn) for 7 continuous days.

### 2.2. Performance and Mortality Records

Raw data derived from measurements taken by the farm’s staff as part of the routine schedule were processed using methods of biostatistics with the Microsoft Excel spreadsheet application. Percent mortality was calculated by dividing the number of birds that died on a day by the number of birds that were alive that day, whereas the Livability percentage was calculated by subtracting the mortality percentage from 100. Feed conversion ratio (FCR) was calculated by using the following formula: FCR = Feed Intake (g)/Weight Gain (g). The European Production Efficiency Factor (EPEF) was calculated according to the formula EPEF = {[Livability (%) × Live weight (Kg)]/[Age(d) × FCR]} × 100 [31]. In addition, the economic efficiency of the farm flocks was calculated by computing the feed cost (€)/live weight (kg) and the feed cost (€)/chick [32], knowing that during the outbreak, the cost of the basal diets was 0.36 €/kg.

### 2.3. Postmortem Examination

Dead (*n* = 30), diseased (*n* = 15), and clinically healthy (*n* = 10) chickens were submitted to the Unit of Avian Medicine, Department of Veterinary Medicine, Aristotle University of Thessaloniki for further examination. At that time, the birds were 23 days old and had not been subjected to any treatment before. After external examination, live chickens were euthanized by carbon dioxide and embedded in disinfected solution (1% Virkon S) before proceeding to necropsy.

### 2.4. Microbiological Investigation

Samples from the liver and spleen were aseptically collected and cultured in commercial 5% sheep blood agar (BD™ Columbia Agar with 5% Sheep Blood) and MacConkey Agar (Merck KGaA, Darmstadt, Germany) following incubation under aerobic, anaerobic (Thermo Scientific™ Oxoid™ AnaeroGen™ 2.5 L Sachet), and microaerobic conditions (Thermo Scientific™ Oxoid™ CampyGen™ 2.5 L Sachet) for 48 h.

### 2.5. Parasitological Investigation

Smears of intestinal content and mucosa were performed for parasitological analysis with direct microscopic examination.

### 2.6. Histopathological Investigation

Liver samples were collected from birds of each Flock (Flock A–C), whereas various tissue samples were taken from affected birds when macroscopic lesions were recorded (Flock A). Samples from the liver, kidney, pancreas, bursa of Fabricius, thymus, spleen, bone marrow, and skeletal muscles were fixed in 10% neutral buffered formalin, processed, and embedded in paraffin. Sections were cut at 4 to 5 μm, mounted on glass slides, and stained with hematoxylin and eosin (H–E) for microscopic examination.

### 2.7. Serology

Blood samples were randomly collected at slaughter age (42 days old), from 25 birds of each flock, to investigate the antibody response of birds to IBDV, CAV, and FAdV. Samples were centrifuged at 3000× *g* for 4 min, and serum was obtained and stored at −20 °C for further serological examination.

For the detection of the FAdV antibodies, a commercial Elisa Kit (Fowl adenovirus Group 1-Antibody test kit, BioChek B.V., Reeuwijk, Netherlands) was used. This kit included a non-specific serotype-common group antigen by 12 serotypes and therefore provided a broad spectrum for detection. According to the technical manual of BioChek, a house was considered FAdV positive if the MT/N ratio (where MT is the mean titer of each bird and N the number of samples) for the sampled birds was >6000 and negative if the MT/N ratio was <6000.

Indirect Elisa commercial kits were also used to detect antibodies to IBDV (Infectious Bursal Disease Virus- Antibody test kit, BioChek, Reeuwijk, The Netherlands) and CAV (Chicken Anemia Virus-Antibody test kit, BioChek Reeuwijk, The Netherlands), according to the instructions of manufacture. For the BioChek CAV Elisa kit, a house was considered CAV positive if the MT/N ratio was >5000 and negative if the MT/N ratio was <5000. The BioChek IBD ELISA kit is suitable for differentiation between live, inactivated, and/or recombinant vaccinated flocks and field infections within the same kit. MTs ranged from 5000–14,000 and were considered a reliable value suggestive of IBDV vaccine (CEVAC^®^ TRANSMUNE IBD, Ceva Animal Health Ltd.) used for vaccinating the broiler flocks in this study.

### 2.8. Toxicology

Feed samples were collected from all diets (starter, grower, and finisher) used during the rearing period. Mycotoxin analysis of feed was performed by LC/ MS-MS following the method of Li et al. [33] for fumonisins detection and Ren et al. [34] for the rest of the toxins, as described by Tsiouris et al. [35]. The results were interpreted as the detected mycotoxins levels/detection limit level. Detection limits were 0.5 μg/kg for AFB_1_, AFB_2_, AFG_1_, and AFG_2_; 1 μg/kg for OTA, T-2, HT-2, and DAS; and 10 μg/kg for ZEN, DON, FB_1_, and FB_2_.

### 2.9. Molecular Investigation

Pooled liver samples from each poultry flock were collected during post-mortem examination and stored at −80 °C until further investigation in the lab. After defrosting, a tissue sample of 0.2–0.4 gr was taken and mixed with 2 mL of phosphate-buffered saline (PBS). The blend was macerated using a rotor homogenizer. Cellular remains were eliminated by centrifugation at 3000× *g* for 15 min, and the total DNA was purified using the DNeasy Blood and Tissue Kit (Qiagen, Hilden, Germany) according to the instructions of the manufacturer. Real-time PCR using a Light Cycler 2.0 (Roche Applied Science, Penzberg, Germany) thermocycler was performed. The primer pair was the 52K-fw/52K-rv, which was previously tested as suitable for the detection of all FAdV species and serotypes [36]. Each 25 μL reaction mixture contained 50–200 ng of purified DNA from each sample, 0.7 μM (final concentration) of each primer (52K-fw and 52K-rv) [36], and HotStart-ITSYBR Green qPCR mixture (Affymetrix, Santa Clara, CA, USA). The amplification conditions included initial denaturation at 95 °C for 5 min, 40 cycles of denaturation at 95 °C for 5 s and annealing/extension at 60 °C for 10 s, and a last melting step between 60 and 95 °C for the confirmation of PCR product Tm, as previously reported [36]. Data analysis was performed using the LightCycler 4.1 software package (Roche Applied Science), automatically adjusting the cycle threshold (CT) value.

Partial sequences were obtained by PCR amplification and Sanger sequencing, targeting the hexon. The PCR reaction was performed with 20 pmol of each primer from Mittal et al. [37] (fwd primer 5’-CAARTTCAGRCAGACGGT-3’ and rev primer 5’-TAGTGATGMCGSGACATCAT-3’), 200 µM dNTPs mix, 1.5 mM MgCl_2_, and 2.5U Taq DNA polymerase. The PCR program consisted of an initial denaturation step at 94 °C for 5 min followed by 35 cycles of denaturation at 94 °C for 1 min, annealing at 58 °C for 1 min, and extension at 72 °C for 1 min with a step of final extension at 72 °C for 10 min. The PCR products, sizing approximately 900 bp, were loaded on a 1% agarose gel for verification. Finally, Sanger sequencing was performed using the forward primer (GATC Biotech, Konstanz, Germany).

The nucleotide sequences of the FAdV isolates were aligned with homologous sequences via the Clustal W method using the Lasergene software (DNASTAR, Madison, WI, USA). A phylogenetic tree for the hexon gene was constructed using MEGA 7.0 software by the neighbor-joining method with 1000 bootstrap replicates. A total of 42 reference strains were used in this study and are summarized in Table S1.

The obtained nucleotide sequence was then compared to other GenBank sequences using the BLAST database (http://www.ncbi.nlm.nih.gov/BLAST/, accessed on 15 March 2021).

## 3. Results

### 3.1. Performance and Mortality Records

**Figure 2:**
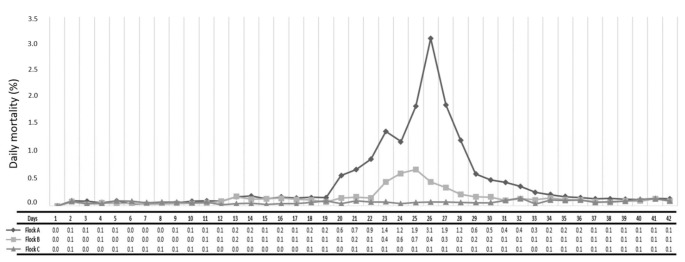
A curve of the daily mortality recorded in each of the three flocks (Flock A–C) of the farm over the rearing period. Daily measurements represent the number of dead birds divided by the population at risk.

In this IBH outbreak, a total of 5709 birds died, and the cumulative mortality was 11.21%. However, as revealed in Figure 2, the clinical manifestation of the disease differed among the flocks.

Daily mortality of Flock A increased exponentially, starting at 20 days of age, making a peak on day 26 (daily mortality 3.1%, or 650 birds), and gradually subsided within 15 days (day 34). During the same period, a shorter but also significant increase in daily mortality was recorded in Flock B, starting at day 23, making a peak on day 25 (daily mortality 0.7%, or 144 birds), and ceasing within 6 days (day 29). However, in Flock C, daily mortality remained stable at low levels during the rearing period. Finally, at the end of the rearing period, the cumulative mortality was 17.4% for Flock A (3791/21,800 birds), 6.1% for Flock B (1322/21,800 birds), and 2.9% for Flock C (596/20,400 birds). In accordance, Livability was computed at 82.60% for Flock A, 93.90% for Flock B, and 97.08% for Flock C. Calculating the feed consumption per bird and the average weight of birds at slaughter age, the FCR index was increased in Flock A (2.07), followed by Flock B (1.90) and Flock C (1.85). The EPEF of Flock A was lower (245) compared to those of Flock B (307) and Flock C (341).

The economic efficiency of each of the farm’s flocks was calculated based on the feed cost, final body weight, and mortality data without including expenses of treatments and the biosecurity measures, applied to the farm in general. Flock A, which was mostly affected, consumed a total of 96.42tn feed (34,711.2 €), reaching a final live mass of 46.49tn. Flock B consumed a total of 104.48tn (37,612.8 €) feed, ending at a live mass of 54.86tn, whereas Flock C consumed a total of 100.00tn (36,000.0 €) feed, reaching a final live mass of 54.08tn. Finally, the feed cost/kg live weight (€/kg) was 0.06 €/kg higher for Flock A (0.75 €/kg), which was mostly affected, compared to Flock B (0.69 €/kg), and 0.08 € /kg higher when compared with that of Flock C (0.67 €/kg). At the end of the rearing period the feed cost/chick (€/chick) was computed at 1.93 €/chick for Flock A, 1.84€/chick for Flock B, and 1.82 €/chick for Flock C.

### 3.2. Postmortem Examination

**Figure 3:**
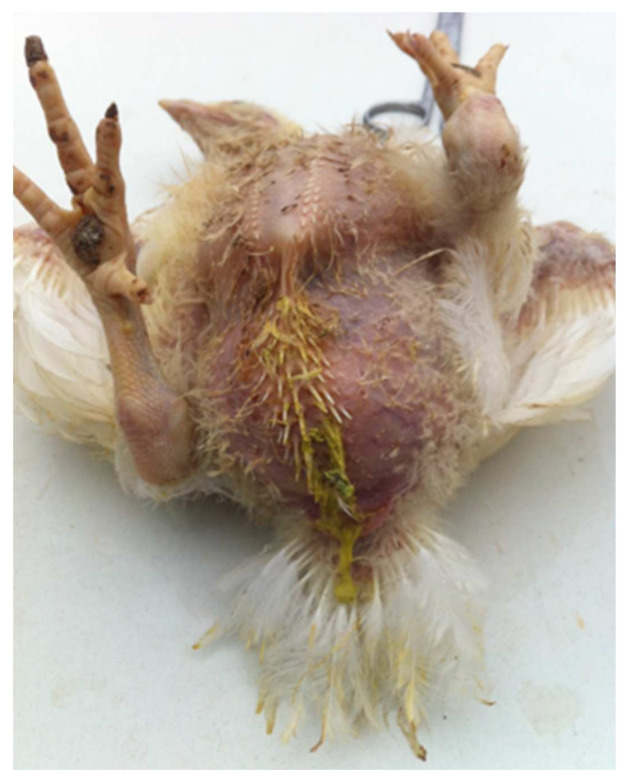
A dead 23-day-old broiler chicken from Flock A. During post-mortem examination, the presence of yellowish mucoid diarrhea, soiling the feather around cloaca, was visible. In addition, dermatitis lesions including hock marks and ulcers can been seen on the legs of the bird.

**Figure 4:**
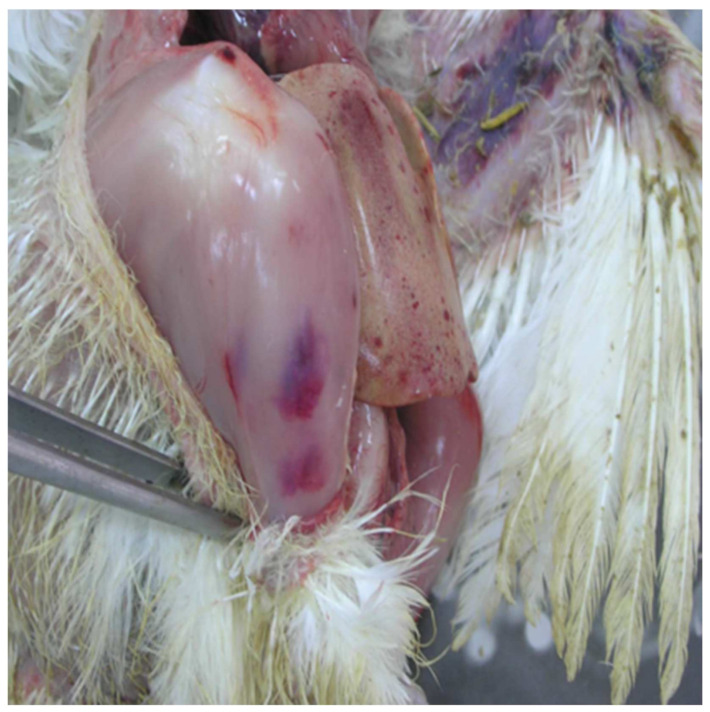
Gross-pathology findings on a 23-day-old broiler chicken, which was submitted for postmortem examination in the Unit of Avian Medicine, Thessaloniki, Greece during the IBH outbreak. The presence of hemorrhages on the skeletal muscles of the bird is visible, whereas, the liver dominates in the visceral capacity with the lobes of the organ appearing swollen with a marble-like pattern ranging from pale and yellowish to red-dark.

**Figure 5:**
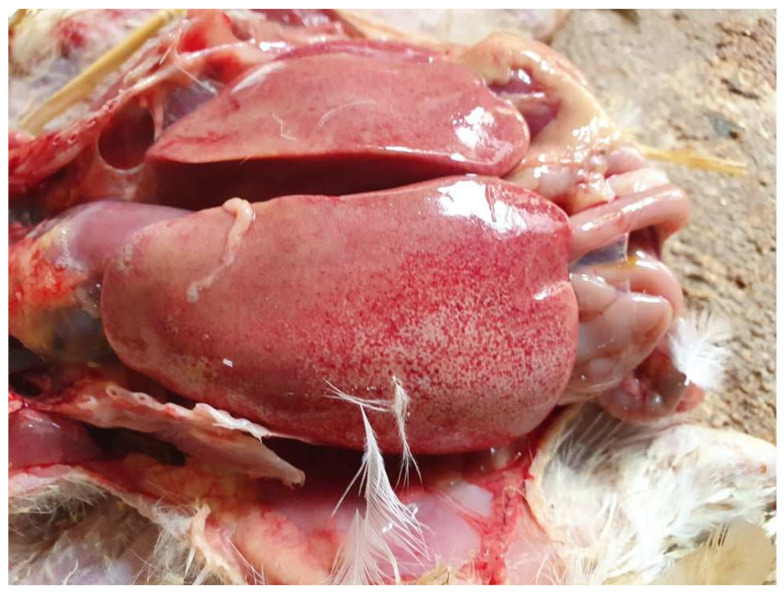
Post-mortem examination of a 23-day-old broiler chicken from the Flock A. The liver dominates in the visceral capacity with the lobes of the organ appearing swollen with a marble-like pattern ranging from pale and yellowish to red-dark.

**Figure 6:**
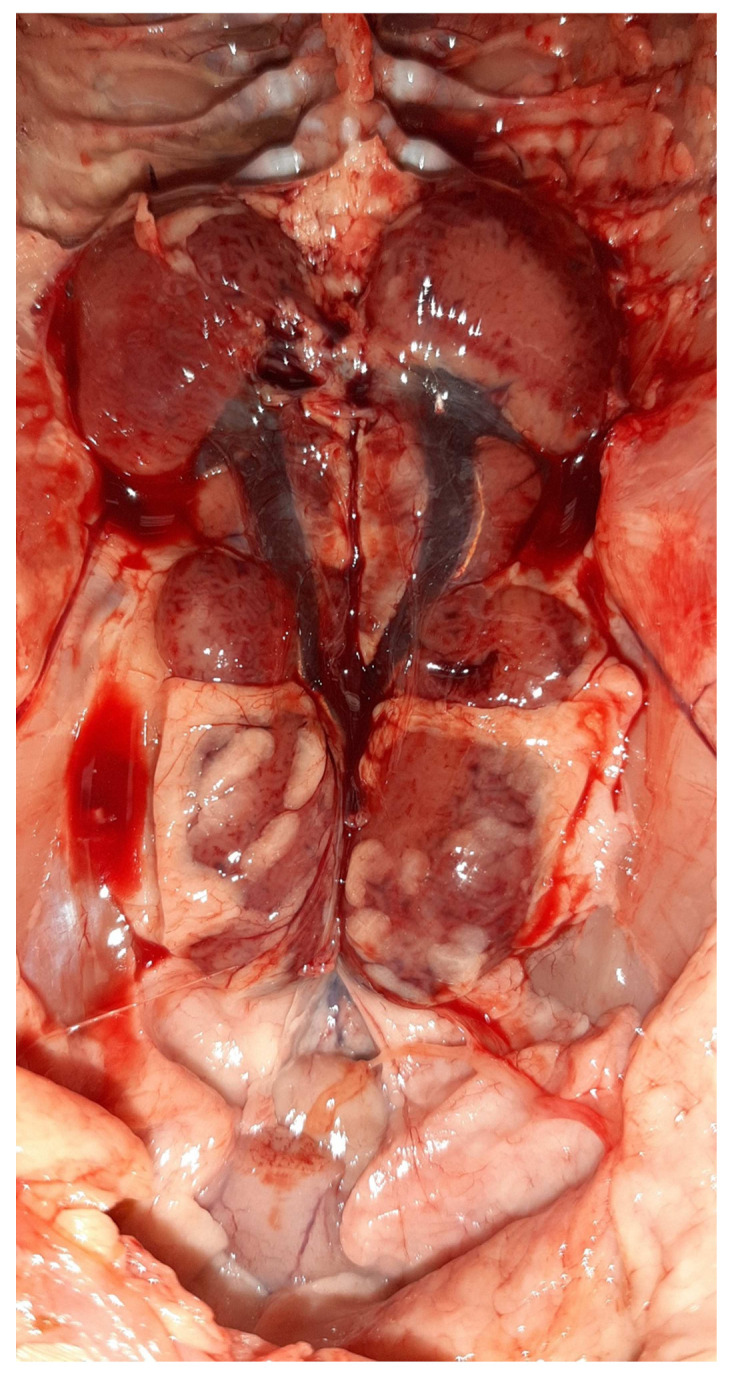
The kidneys of an affected bird are pictured oedematous and mottled, enlarged with urates accumulation in the ureters.

**Figure 7:**
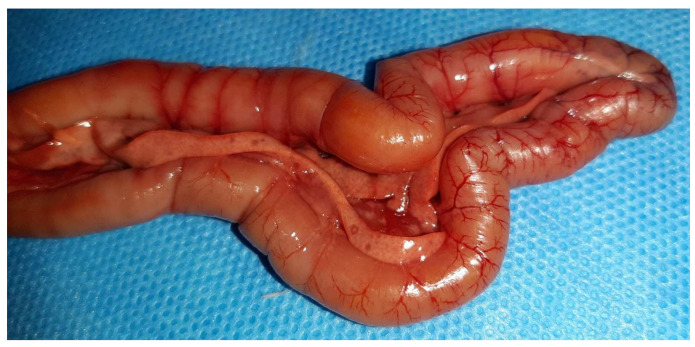
Gross-pathology findings on the pancreas of a 23-day-old broiler chicken, which was submitted for postmortem examination in the Unit of Avian Medicine, Thessaloniki, Greece during the IBH outbreak. The presence of necrotic spots and hemorrhage or/and congestion are visible on the surface of the organ.

On necropsy, most of the chickens had yellowish mucoid diarrhea soiling the feather of the cloaca (Figure 3). Icteric skin and subcutaneous fat were notable in affected birds, while petechial hemorrhages on leg and breast muscles were rarely recorded (Figure 3). During evisceration, the liver dominated the visceral capacity with the lobes of the organ appearing swollen with a marble-like pattern ranging from pale and yellowish to red-dark (Figure 4 and Figure 5). Similarly, kidneys were pale, oedematous, and mottled, enlarged with urates accumulation in the ureters (Figure 6). In many birds, the pancreas was also affected and was slightly inflamed with necrotic foci spotting the surface of the organ (Figure 7). Finally, some birds presented a moderately atrophic Bursa of Fabricius and thymus.

### 3.3. Microbiological Investigation

Pathogenic bacteria were not isolated.

### 3.4. Parasitology

The direct microscopic examination of the intestinal smears revealed the presence of a small number (+) of *Eimeria* spp. oocysts.

### 3.5. Histopathology

**Figure 8:**
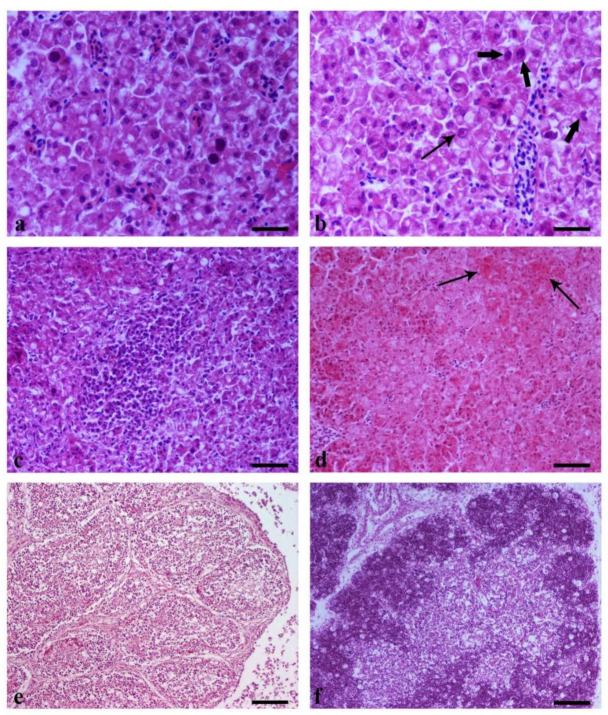
(**a**) Liver. Fatty degeneration and necrosis of hepatocytes and presence of intensely basophilic intranuclear inclusions bodies. H-E. Bar, 25 μm. (**b**) Liver. Fatty degeneration and necrosis of hepatocytes. Intranuclear inclusion bodies, eosinophilic surrounded by light halo (thin arrow) and basophilic (thick arrows), are obvious. H-E. Bar, 25 μm. (**c**) Liver. Marked centrally located inflammatory cell infiltration consists of lymphocytes, macrophages, plasmacytes, and heterophils. Hepatocytes show fatty degeneration and/or necrosis as well as basophilic intranuclear inclusions bodies. H–E. Bar, 50 μm. (**d**) Liver. Hepatocyte vacuolar degeneration is prominent, accompanied by areas of coagulative hepatocellular necrosis (arrows). H–E. Bar, 50 μm. (**e**) Bursa. Atrophy of lymphoid follicles with intense depletion of lymphocytes both in the cortex and medulla. H–E. Bar, 100 μm. (**f**) Thymus. Lymphoid depletion is prominent mostly in the cortex of the thymus, and interlobular spaces present mild edema and vascular distention. Note the scattered clear areas located mostly in the cortex, some of which contain small dark nuclei, characteristic of apoptosis in lymphoid organs. H–E. Bar, 100 μm.

**Figure 9:**
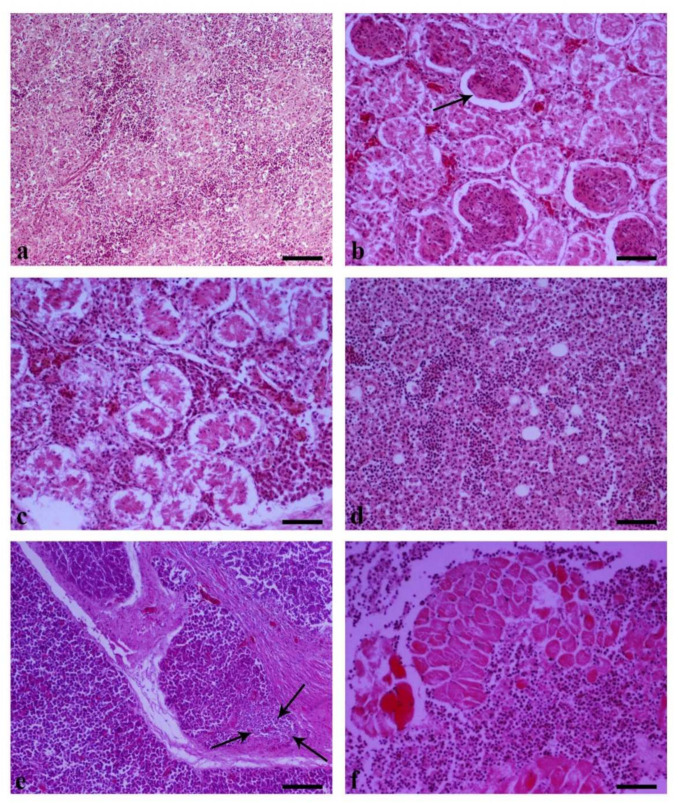
(**a**) Spleen. Intense depletion of lymphoid follicles characterized by prominence of reticular cells in the absence of lymphocytes in the germinal center. H–E. Bar, 100 μm. (**b**) Kidney. Renal glomeruli are hypercellular due to increase in mesangial cell number (mesangio proliferative glomerulonephritis). Note a glomerulus with segmental glomerulosclerosis (arrow). Tubular epithelial necrosis, interstitial hyperemia, and hemorrhage are also observed. H–E. Bar, 50 μm. (**c**) Kidney. The tubular epithelium is characterized by necrosis, and in the interstitial connective tissue are seen aggregates of mononuclear inflammatory cells. H–E. Bar, 50 μm. (**d**) Bone marrow. Mild depletion. Sparse cells throughout the bone marrow present nuclear pyknosis and, more often, fragmentation suggesting necrosis of bone marrow. H–E. Bar, 50 μm. (**e**) Pancreas. Pancreatic lobules present loss of their architecture (absence of acini). The interlobular connective tissue is intensely increased, consisting of fibroblasts and collagen fibers, and is infiltrated by a few mononuclear cells. Additionally, a lobule reveals a large aggregate of mononuclear cells to its lower right side (arrows) H–E. Bar, 100 μm. (**f**) Skeletal muscle. Degeneration and necrosis of muscle fibers and infiltration of the perimysium and endomysium with mononuclear cells and heterophils. H–E. Bar, 50 μm.

Liver samples from birds collected from Flock A and B showed prominent vacuolar degeneration, areas of coagulative hepatocellular necrosis with intranuclear, large, deeply basophilic inclusion bodies characteristic of adenovirus infection (Figure 8a–c). Multiple samples from the liver of one bird from Flock C revealed the same pattern with those of Flocks A and B. However, inclusion bodies were seldom observed (Figure 8d). In the Bursa, the follicular cortex and medulla were depleted of lymphoid cells (Figure 8e). Thymic atrophy with lymphoid depletion was noted, mainly in the cortex, accompanied with mild edema of the interlobular spaces and vascular distention (Figure 8f). Some mononuclear inflammatory cells and hemorrhages were noted throughout the medulla. Severe depletion of lymphoid follicles also characterized the spleen. Loss of lymphoid cells led to the prominence of reticular cells in the germinal center (Figure 9a). Kidneys showed increased glomerular cellularity due to mesangial cell proliferation (mesangioproliferative glomerulonephritis) (Figure 9b). There was observed tubular epithelial necrosis, interstitial hyperemia and hemorrhages, as well as infiltration of the interstitial connective tissue with mononuclear inflammatory cells (Figure 9c). Bone marrow was characterized by mild depletion with sparse cells throughout the bone marrow presenting nuclear pyknosis and, more often, fragmentation, suggesting the necrosis of bone marrow (Figure 9d). Loss of the architecture of pancreatic lobules was observed due to the absence of acini accompanied by intensely increased interlobular connective tissue and the presence of foci of mononuclear cells aggregates suggestive of chronic inflammation (Figure 9e). Finally, examination of skeletal muscles revealed degeneration, the necrosis of muscle fibers and infiltration of the perimysium and endomysium with mononuclear cells, and heterophils (Figure 9f).

(a–c) Flock A + B and (d) Flock C.8.1. Subsection

### 3.6. Serology

Elisa results revealed that FAdV, IBDV, and CAV antibody titers in all flocks were normally distributed. All the flocks were serologically positive for FAdV infection with the MT/N ratio having been calculated for each flock: Flock A: 6926, Flock B: 6523, and Flock C: 6553, *n* = 25. For IBDV the MT/N ratio was also calculated for each flock (Flock A: 7218, Flock B: 7230, and Flock C: 7211, *n* = 25), and results were anticipated to the titers suggested for the vaccination. For CAV, the results were anticipated to the titers suggested for maternal immunity due to the vaccination of the breeders. The MT/N ratio was also calculated for each flock; Flock A: 1408, Flock B: 1418, and Flock C: 1414, *n* = 25.

### 3.7. Toxicology

TLC chromatography revealed that mycotoxins were under detection limits in all starter, growing, and finishing diets.

### 3.8. Molecular Investigation

Three FAdVs strains were detected from Flock A, B, and C, respectively. However, the nucleotide sequences of the FAdVs isolates were completely identical (100%). The analysis at the tree involved 43 nucleotide sequences in total (Table S1).

**Figure 10:**
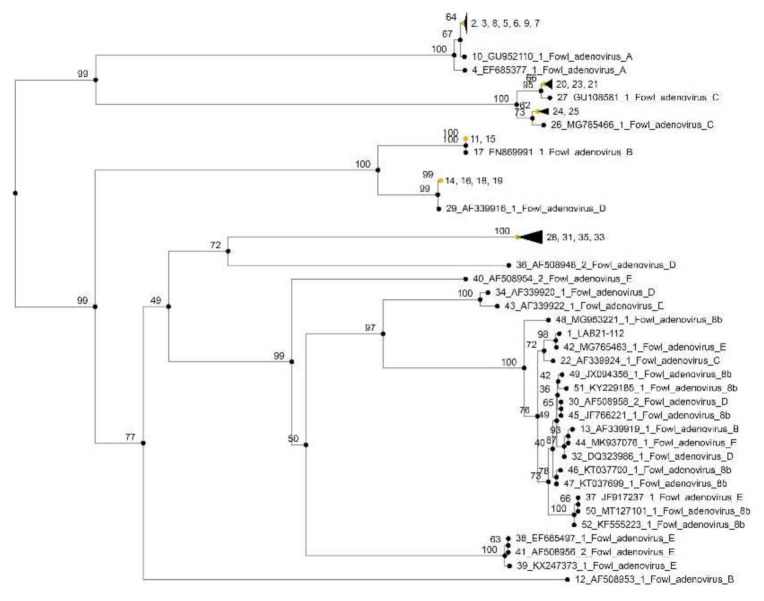
Phylogenetic analysis of the isolated strain (LAB21-112) based on hexon L1. Strain LAB21-112 was sequenced in this study; the sequences of the other strains were downloaded from GenBank. The phylogenetic tree was generated by MEGA 7.0 software.

According to phylogenetic analysis (Figure 10) based on the hexon gene with available sequences from GenBank, FAdVs were classified as FAdV-E serotype 8b, showing almost equal nucleotide identities (99.76%) with the Peruvian isolate (Accession n. MG765463), which belongs to species E, serotype 8b. Interestingly, the isolate LAB21-112 revealed low similarities (<90%) with the other FAdV-E isolates. More specifically, the isolate had identity 86.26% with that from Canada (Accession n. EF685497), 86.26% for FAdV-8 “TR59” strain (Accession n. AF508956), 90.61% for FAdV-7 isolate (Accession n. AF339922), and 85.27% for the European FAdV-6 serotype (Accession n. AF508954). Surprisingly, a high similarity (98.45%) with isolate (Accession n. AF 339924), which belongs to FAdV of species C and serotype 10, was also noted.

We also compared the nucleotide sequence of the isolate LAB21-112 with other GenBank sequences using the BLAST database (http://www.ncbi.nlm.nih.gov/BLAST/. accessed on 15 March 2021). Interestingly, the isolate LAB21-112 revealed high similarities (>99%) with other FAdV-E isolates previously reported in Greece (Accession n. MK572862), Germany (Accession n. MK572863), Hungary (Accession n. MK572858), China (Accession n, MG712775, KU981150, KY426984, MG547385, MF573922, MF573919, MF573907, MF577036), and Peru (Accession n. KX258422 MG765468, MG765461, MG765463, MG547388).

## 4. Discussion

IBH is a viral disease of poultry with considerable significance since its first report in the 1960s [1,4,8,9,20]. In many regions worldwide, the disease has been endemic and become of dominant concern for young broilers of 3–5 weeks of age [4,8,38,39]. In affected areas, increased biosecurity and vaccination programs using autogenous vaccines were partially effective. The lack of cross-species protection resulted in new serotypes to prevail among vaccinated flocks in affected areas [24,40], whereas the resistance of FAdVs to the commonly used disinfectants [41] makes it difficult to control the spread of the pathogen. In Greece, neither broiler breeder nor broiler chicken flocks are vaccinated against FAdVs and therefore are vulnerable to FAdVs infections. In addition, there is scarce information about the virulence of the circulating FAdVs and the role of predisposing factors involved to their clinical manifestation in the field.

In our case, affected birds were at 20 days of age, exhibiting nonspecific clinical signs other than suddenly increased daily mortality. In classical IBH cases, clinical signs and mortality peak after 3–4 days and usually return to normal on the 6th day of the outbreak [1,13,14]. Rarely, clinical signs and mortality can persist for more than 2 weeks, resulting in a dramatic effect on the production [38]. In our case, clinical signs and increased daily mortality (%) lasted for 15 days in Flock A (16.81%) and 6 days in Flock B (6.1%), whereas Flock C was clinically unaffected. FCR, as well as EPEF, were adversely affected mainly in Flock A, and less in Flock B and C following the diminutive pattern of the clinical course of the disease among the flocks. Finally, the producer reported a remarkable increase in the production cost, mainly for Flock A, as a result of the increased mortality and growth retardation that persisted until slaughter. The punctual economic impact is difficult to determine but, according to an epidemiological survey from Mississippi, broiler meat production cost was $0.0058/kg more expensive to produce when IBH occurred [7].

A necropsy and histopathological examination on affected birds revealed that liver lesions were predominant, all in terms of classical IBH cases. The liver was enlarged, pale, and friable with a histopathological picture of multifocal inflammation and necrosis, alongside the presence of characteristic large basophilic inclusion bodies in hepatocytes. Inclusion bodies may be found in the cells of other tissues of poultries as a result of FAdV or another viral replication [42,43]. However, the detection of eosinophilic or basophilic inclusion bodies in hepatocytes are pathognomonic in terms of FAdVs and could lead to the diagnosis [17,36]. Basophilic inclusions are mainly formatted by virus particles, whereas eosinophilic usually consist of degenerated or fibrillar granular material in cells dying from the viral infection [44]. In our case, most inclusion bodies were basophilic, whereas eosinophilic inclusions were rarely noted. Basophilic inclusions are formed first, and then the eosinophilic inclusions are produced. Thus, in our case, the basophilic inclusion formation in liver samples from Flock A and B could be attributed to a severe and rapid infection with FAdV of these flocks. Continuing on, the quite small number of hepatocellular inclusions, the absence of lesions in the rest of the organs examined, and the absence of clinical signs in birds sampled from Flock C could be attributed to a very early or a very late stage of infection or to an infection with another non-pathogenic FAdV serotype [15]. However, in our case, PCR analysis of the hexon gene revealed that isolates from all the flocks shared equal nucleotide identity (100%).

After infection, FAdVs primarily replicate in the enteric or respiratory epithelium following viraemia transfer to their target organs. Previous investigators revealed that FAdVs can replicate in the tubular epithelium of kidneys, resulting in proliferative glomerulonephritis [45], which was a prevailing finding in our case as well. However, lesions in the pancreas have been currently described by some reports, revealing a new feature to FAdV infection and IBH pathology [17,39,46,47]. In our case, a loss of the architecture of pancreatic lobules was observed, due to the absence of acini accompanied by intensely increased interlobular connective tissue. In addition, the foci of mononuclear cell aggregates were observed, suggesting a chronic inflammation. However, pancreatitis was never the main focus of previous investigations, and the detrimental effects on the function of the pancreas were not studied in detail.

The mechanism of FAdV pathogenicity is not fully elucidated. However, under in-field conditions, IBH outbreaks are associated with a variety of diseases including bacterial infections, viral agents, mainly those historically involved (CAV and IBDV), parasites, and mycotoxins [5,7,18,19,20]. The above agents could act as predisposing factors or as secondary co-infectants enhancing the exacerbation of the disease. However, the microbiological and the parasitological investigation revealed that in this outbreak, neither bacterial nor parasitic agents were involved. Furthermore, the TLC chromatography revealed that mycotoxins were not associated with the current outbreak since they were under detection limits in all animal diets.

Broiler breeders in Greece are normally vaccinated against CAV to prevent vertical transmission of the virus and provide protection to their progenies for the clinical and subclinical form of the disease. Similarly, live and killed IBD vaccines are applied in both breeder and broiler flocks in the country. Thus, it is not surprising that IBDV and CAV antibody titers were at the expected levels in all flocks excluding the predisposing role of these agents for the present IBH outbreak. This is in accordance with previously published reports, which present FAdVs as primary agents in IBH [21,22,23,24]. Hence, the mild loss of the lymphoid cells in the thymus and cloacal bursa of the IBH-affected chickens in the present case are likely associated with the involvement of this primary lymphoid organ in the pathology of FAdV infection [48]. In addition, the depletion of the cells that occurred in the bone marrow could be attributed to the FAdV effect in the hematopoietic system of birds, a fact also noted by other researchers [16].

In the current study, phylogenetic analyses of the hexon regions of isolated FAdV stains revealed that they were 99.86% identical with the Greek isolate (Accession n. MK572862), which belongs to FAdVs of species E and is part of a homogeneous clade composed by Greek-only sequences, sampled in 2013 [12,29]. These findings are in accordance with Frantzo et al. [29], who reported that 85.29% of strains involved in IBH outbreaks in the region of Epirus in Greece were of species E. Hence, the scenario that FAdVs of species E are dominant in Greece and can cause natural clinical diseases in chickens may be suggested.

FAdV isolates of this study were 98.76% to the Peruvian isolate (Accession n. MG765463), which belongs to species E and serotype 8b. During the last decade, FadV 8b has been associated with outbreaks of IBH in Poland [27], Spain [9], Slovenia [25], Turkey [38,39], Canada [49], Korea [5], Malaysia [50], South Africa [51], Peru [52], Iran [53], Brazil [54], and China [8].

The global chicken and egg trade can increase the chance for introducing viral pathogens, including FAdVs, to countries [55], whereas wild birds may provide an additional route as the cross-species transmission is already evident [56]. In FAdV epidemiology, both vertical and horizontal transmission are involved [13]. Although vertical transmission could be advocated in several outbreaks, the horizontal one was most likely involved in this outbreak. Contaminated vehicles, personnel, equipment, and biosecurity gaps could induce and spread the infection to the farm [57]; however, it is difficult to determine the actual route. From the history of the farm, the personnel started daily routine management from Flock A, to B, to C. We suggest that in our case, Flock A was initially infected, as it showed higher morbidity and mortality, and therefore was a route of transmission to the rest of the flocks. This scenario could support the diminutive manifestation of the disease from Flock A to C, however other factors, for those which were studied (e.g., house microenvironment, orientation etc.), could also be implemented.

Controlling horizontal transmission of the FAdVs may be achieved by strict biosecurity measures. Infected birds develop a viremia and increase shedding of the virus after 2–3 days in high loads with feces [58]. Lateral spread is possible because the virus is present in droppings and is resistant to inactivation [41]. Circumstantial evidence exists for spread by personnel and transport; therefore, hygienic precautions are required [27]. In our instance, the suspicion of IBH prompted us to apply strict biosecurity measures among the flocks for the rest of the rearing period, as well as in the farm generally. In addition, we suggested altering the daily routine management from Flock C, to B, to A. Maybe, this handling contributed to maintain Flock C clinically unaffected.

Since there is no treatment for IBH, a commercial poultry hepatoprotectant, a blend of silymarin, amino-acids, vitamins, and electrolytes, was administrated via drinking water to stimulate the liver function and the immune system of the affected birds, as previously proposed by Venne and Chorfi [59].

Adenoviruses are generally tolerant to heat and pH changes, as well as to the majority of commercial disinfectants [41]. Thus, district disinfection with disinfectants containing iodophor or aldehyde was suggested to reduce the possibility of the contamination of the next broiler flocks that are going to be placed in the farm. Furthermore, a minimum “chicken-free” downtime of 3 weeks was recommended to ensure the reduction of the viral load in the farm. Finally, the use of vectored IBD vaccine was proposed in order to reduce the immunosuppressive effect of attenuated and/or immune complex vaccines. Until today, the farm has not been challenged again with IBH; however, biosecurity and management are strictly supported by the flock’s staff.

## 5. Conclusions

The existence of multiple FAdV serotypes and the lack of interspecies cross-protection make the control of IBH extremely difficult. Thus, more epidemiological information should be collected, and a broad-spectrum FAdV vaccine is needed for the prevention of IBH. In Greece, there is a shortage of data about the prevalence of the disease in commercial poultry flocks. To the best of our knowledge, this study reported for the first time the implication of FAdV-8b in an outbreak of IBH in broilers in the country. In addition, with our study, the FAdV-8b has strong evidence to be a primary pathogen, causing disease and high mortality without correlation with IBDV, CAV, and mycotoxins. With this study, we aim to provide more epidemiological data about the distribution of FAdVs in commercial flocks. However, further work is needed to determine the virulence of this FAdV isolate.

## Data Availability

None of the data presented were deposited in an official repository.

## Supplementary Materials

The following supporting information can be downloaded at: https://www.mdpi.com/article/10.3390/vetsci9040160/s1. Table S1: Available epidemiological data, for the total of 42 reference strains which were used for the construction of the phylogenetic tree in this study.

## References

[1] Schachner A., Matos M., Grafl B., Hess M. (2018). Fowl adenovirus-induced diseases and strategies for their control—A review on the current global situation. Avian Pathol..

[2] McFerran J.B., Smyth J.A. (2000). Avian adenoviruses. Rev. Sci. Tech..

[3] Helmboldt C.F., Frazier M.N. (1963). Avian hepatic inclusion bodies of unknown significance. Avian Dis..

[4] Chen L., Yin L., Zhou Q., Peng P., Du Y., Liu L., Zhang Y., Xue C., Cao Y. (2019). Epidemiological investigation of fowl adenovirus infections in poultry in China during 2015–2018. BMC Vet. Res..

[5] Choi K.S., Kye S.J., Kim J.Y., Jeon W.J., Lee E.K., Park K.Y., Sung H.W. (2012). Epidemiological investigation of outbreaks of fowl adenovirus infection in commercial chickens in Korea. Poult. Sci..

[6] Kiss I., Homonnay Z.G., Mató T., Bányai K., Palya V. (2021). Research Note: An overview on distribution of fowl adenoviruses. Poult. Sci..

[7] Sentíes-Cué C.G., Wills R.W., Stayer P.A., Burleson M.A., Magee D.L. (2010). Epidemiology and effect on production parameters of an outbreak of inclusion body hepatitis in broilers. Avian Dis..

[8] Niu Y., Sun Q., Zhang G., Sun W., Liu X., Xiao Y., Shang Y., Liu S. (2018). Epidemiological investigation of outbreaks of fowl adenovirus infections in commercial chickens in China. Transbound. Emerg. Dis..

[9] Oliver-Ferrando S., Dolz R., Calderón C., Valle R., Rivas R., Pérez M., Biarnés M., Blanco A., Bertran K., Ramis A. (2017). Epidemiological and pathological investigation of fowl aviadenovirus serotypes 8b and 11 isolated from chickens with inclusion body hepatitis in Spain (2011–2013). Avian Pathol..

[10] Meulemans G., Couvreur B., Decaesstecker M., Boschmans M., Berg T. (2004). Phylogenetic analysis of fowl adenoviruses. Avian Pathol..

[11] Harrach B., Benkö M., Both G.W., Brown M., Davison A.J., Echavarría M., Hess M., Jones M.S., Kajon A., Lehmkuhl H.D., King A.M.Q., Adams M.J., Castens E.B., Lefkowitz E.J. (2012). Family Adenoviridae. Virus Taxonomy: Ninth Report of the International Committee on Taxonomy of Viruses.

[12] Schachner A., Marek A., Grafl B., Hess M. (2016). Detailed molecular analyses of the hexon loop-1 and fibers of fowl aviadenoviruses reveal new insights into the antigenic relationship and confirm that specific genotypes are involved in field outbreaks of inclusion body hepatitis. Vet. Microbiol..

[13] Kichou F., Zro K., Mouahid M., Berrada J. (2019). Emerging and reemerging fowl aviadenovirus infections. Emerging and Reemerging Viral Pathogens: Volume 1: Fundamental and Basic Virology Aspects of Human, Animal and Plant Pathogens.

[14] Hafez H.M. (2011). Avian adenoviruses infections with special attention to inclusion body hepatitis/hydropericardium syndrome and egg drop syndrome. Pak. Vet. J..

[15] Garmyn A., Bosseler L., Braeckmans D., Erum J., Verlinden M. (2018). Adenoviral Gizzard Erosions in Two Belgian Broiler Farms. Avian Dis..

[16] Niczyporuk J.S. (2018). Adenoviruses and Their Diversity in Poultry. Application of Genetics and Genomics in Poultry Science.

[17] Matos M., Grafl B., Liebhart D., Hess M. (2016). The outcome of experimentally induced inclusion body hepatitis (IBH) by fowl aviadenoviruses (FAdVs) is crucially influenced by the genetic background of the host. Vet. Res..

[18] El-Tholoth M., Abou El-Azm K.I. (2019). Molecular detection and characterization of fowl adenovirus associated with inclusion body hepatitis from broiler chickens in Egypt. Trop. Anim. Health Prod..

[19] Mariappan A.K., Munusamy P., Latheef S.K., Singh S.D., Dhama K. (2018). Hepato nephropathology associated with inclusion body hepatitis complicated with citrinin mycotoxicosis in a broiler farm. Vet. World.

[20] Herdt P., Timmerman T., Defoort P., Lycke K., Jaspers R. (2013). Fowl adenovirus infections in Belgian broilers: A ten-year survey. Vlaams Diergeneeskd. Tijdschr..

[21] Gomis S., Goodhope R., Ojkic D., Willson P. (2006). Inclusion body hepatitis as a primary disease in broilers in Saskatchewan, Canada. Avian Dis..

[22] Mirzazadeh A., Asasi K., Mosleh N., Abbasnia M., Abdi Hachesoo B. (2020). A primary occurrence of inclusion body hepatitis in absence of predisposing agents in commercial broilers in Iran: A case report. Iran. J. Vet. Res..

[23] Nakamura K., Mase M., Yamamoto Y., Takizawa K., Kabeya M., Wakuda T., Matsuda M., Chikuba T., Yamamoto Y., Ohyama T. (2011). Inclusion Body Hepatitis Caused by Fowl Adenovirus in Broiler Chickens in Japan, 2009–2010. Avian Dis..

[24] Steer P.A., Ghorashi S.A., Noormohammadi A.H. (2011). Application of high-resolution melting curve analysis for typing of fowl adenoviruses in field cases of inclusion body hepatitis. Aust. Vet. J..

[25] Zadravec M., Slavec B., Krapež U., Kaján G.L., Račnik J., Juntes P., Juršič Cizerl R., Benkõ M., Zorman Rojs O. (2013). Inclusion body hepatitis (IBH) outbreak associated with Fowl adenovirus type 8b in broilers. Acta Vet..

[26] Gaweł A., Nowak M., Ciaputa R., Bobrek K. (2016). Prevalence of inclusion body hepatitis (IBH) in Poland from 2010–2014. Polish J. Vet. Sci..

[27] Niczyporuk J.S., Kozdrun W., Czekaj H., Piekarska K., Stys-Fijol N. (2021). Characterisation of adenovirus strains represented species B and E isolated from broiler chicken flocks in eastern Poland. Heliyon.

[28] Virol A., Niczyporuk J.S. (2017). Molecular characterisation of fowl adenovirus type 7 isolated from poultry associated with inclusion body hepatitis in Poland. Arch. Virol..

[29] Franzo G., Prentza Z., Paparounis T., Tsiouris V., Centonze G., Legnardi M., Catelli E., Tucciarone C.M., Koutoulis K., Cecchinato M. (2020). Molecular epidemiology of fowl adenoviruses in Greece. Poult. Sci..

[30] Tsiouris V., Georgopoulou I., Andreopoulou M., Hess M., Poutachidis T. Inclusion Body Hepatitis: Newest epidemiological data. Proceedings of the 3rd Panhellenic Conference of Reproductive Animals and Food Safety.

[31] Aviagen (2018). Ross 308 Handbook. https://en.aviagen.com/assets/Tech_Center/Ross_Broiler/Ross-BroilerHandbook2018-EN.pdf.

[32] Marcu A., Vacaru-opri I., Dumitrescu G., Petculescu L., Marcu A., Nicula M., Pe I., Dronca D., Kelciov B., Mari C. (2013). The Influence of Genetics on Economic Efficiency of Broiler Chickens Growth. Anim. Sci. Biotechnol..

[33] Li W., Herrman T.J., Dai S.Y. (2010). Rapid determination of fumonisins in corn-based products by liquid chromatography/tandem mass spectrometry. J. AOAC Int..

[34] Ren Y., Zhang Y., Shao S., Cai Z., Feng L., Pan H., Wang Z. (2007). Simultaneous determination of multi-component mycotoxin contaminants in foods and feeds by ultra-performance liquid chromatography tandem mass spectrometry. J. Chromatogr..

[35] Tsiouris V., Tassis P., Raj J., Mantzios T., Kiskinis K., Vasiljević M., Delić N., Petridou E., Brellou G.D., Polizopoulou Z. (2021). Investigation of a novel multicomponent mycotoxin detoxifying agent in amelioration of mycotoxicosis induced by aflatoxin-b1 and ochratoxin a in broiler chicks. Toxins.

[36] Günes A., Marek A., Grafl B., Berger E., Hess M. (2012). Real-time PCR assay for universal detection and quantitation of all five species of fowl adenoviruses (FAdV-A to FAdV-E). J. Virol. Methods.

[37] Mittal D., Jindal N., Tiwari A.K., Khokhar R.S. (2014). Characterization of fowl adenoviruses associated with hydropericardium syndrome and inclusion body hepatitis in broiler chickens. Virus Dis..

[38] Şahindokuyucu İ., Çöven F., Kılıç H., Yılmaz Ö., Kars M., Yazıcıoğlu Ö., Ertunç E., Yazıcı Z. (2020). First report of fowl aviadenovirus serotypes FAdV-8b and FAdV-11 associated with inclusion body hepatitis in commercial broiler and broiler-breeder flocks in Turkey. Arch. Virol..

[39] Cizmecigil U.Y., Umar S., Yilmaz A., Bayraktar E., Turan N., Tali B., Aydin O., Tali H.E., Yaramanoglu M., Yilmaz S.G. (2020). Characterisation of fowl adenovirus (FAdV-8b) strain concerning the geographic analysis and pathological lesions associated with inclusion body hepatitis in broiler flocks in Turkey. J. Vet. Res..

[40] Steer-Cope P.A., Sandy J.R., O’Rourke D., Scott P.C., Browning G.F., Noormohammadi A.H. (2019). Vaccination with FAdV-8a induces protection against inclusion body hepatitis caused by homologous and heterologous strains. Avian Pathol..

[41] Inoue D., Hayashima A., Tanaka T., Ninomiya N., Tonogawa T., Nakazato S., Mase M. (2020). Virucidal effect of commercial disinfectants on fowl adenovirus serotype 1 strains causing chicken gizzard erosion in Japan. J. Appl. Poult. Res..

[42] Bougiouklis P.A., Sofia M., Brellou G., Georgopoulou I., Billinis C., Vlemmas I. (2007). A clinical case of chicken infectious anemia disease and virus DNA detection in naturally infected broilers in Greece. Avian Dis..

[43] Tsiouris V., Mavromati N., Kiskinis K., Mantzios T., Homonnay Z.G., Mato T., Albert M., Kiss I., Georgopoulou I. (2021). A case of infectious laryngotracheitis in an organic broiler chicken farm in Greece. Vet. Sci..

[44] McFerran J.B., Adair B.M.C. (1977). Avian adenoviruses—A review. Avian Pathol..

[45] Wilson F.D., Wills R.W., Senties-Cue C.G., Maslin W.R., Stayer P.A., Magee D.L. (2010). High incidence of glomerulonephritis associated with inclusion body hepatitis in broiler chickens: Routine histopathology and histomorphometric studies. Avian Dis..

[46] Hess M. (2017). Commensal or pathogen—A challenge to fulfil Koch’s Postulates. Br. Poult. Sci..

[47] Radwan M.M., El-deeb A.H., Mousa M.R., El-Sanousi A.A. (2019). First report of fowl adenovirus 8a from commercial broiler chickens in Egypt: Molecular characterization and pathogenicity. Poult. Sci..

[48] Steer P.A., Sandy J.R., O’Rourke D., Scott P.C., Browning G.F., Noormohammadi A.H. (2015). Chronological analysis of gross and histological lesions induced by field strains of fowl adenovirus serotypes 1, 8b and 11 in one-day-old chickens. Avian Pathol..

[49] Dar A., Gomis S., Shirley I., Mutwiri G., Brownlie R., Potter A., Gerdts V., Tikoo S.K. (2012). Pathotypic and molecular characterization of a fowl adenovirus associated with inclusion body hepatitis in Saskatchewan chickens. Avian Dis..

[50] Juliana M.A., Nurulfiza I., Hair-Bejo M., Omar A.R., Aini I. (2014). Molecular characterization of fowl adenoviruses isolated from inclusion body hepatitis outbreaks in commercial broiler chickens in Malaysia. Pertanika J. Trop. Agric. Sci..

[51] Maartens L.H., Joubert H.W., Aitchison H., Venter E.H. (2014). Inclusion body hepatitis associated with an outbreak of fowl adenovirus type 2 and type 8b in broiler flocks in South Africa. J. S. Afr. Vet. Assoc..

[52] Izquierdo-Lara R., Calderón K., Chumbe A., Montesinos R., Montalván Á., González A.E., Icochea E., Fernández-Díaz M. (2016). Complete genome sequence of fowl aviadenovirus serotype 8b isolated in South America. Genome Announc..

[53] Morshed R., Hosseini H., Langeroudi A.G., Fard M.H.B., Charkhkar S. (2017). Fowl Adenoviruses D and e Cause Inclusion Body Hepatitis Outbreaks in Broiler and Broiler Breeder Pullet Flocks. Avian Dis..

[54] Torre D., Nuñez L.F.N., Santander Parra S.H., Astolfi-Ferreira C.S., Piantino Ferreira A.J. (2018). Molecular characterization of fowl adenovirus group I in commercial broiler chickens in Brazil. Virus Dis..

[55] Radin J.M., Shaffer R.A., Lindsay S.P., Araneta M.R.G., Raman R., Fowler J.H. (2017). International chicken trade and increased risk for introducing or reintroducing highly pathogenic avian influenza A (H5N1) to uninfected countries. Infect. Dis. Model..

[56] Niczyporuk J.S., Kozdruń W., Czekaj H., Styś-Fijoł N., Piekarska K. (2020). Detection of fowl adenovirus D strains in wild birds in Poland by Loop-Mediated Isothermal Amplification (LAMP). Vet. Res..

[57] Hess M., Swayne D.E., Glisson J.R., McDougald L.R., Nolan L.K., Suarez D.L., Nair V. (2013). Aviadenovirus infections. Diseases of Poultry.

[58] Yugo D.M., Hauck R., Shivaprasad H.L., Meng X.J. (2016). Hepatitis Virus Infections in Poultry. Avian Dis..

[59] Venne D., Chorfi Y. Blood biochemical changes in birds with inclusion body hepatitis and the effect of supportive treatments during outbreaks. Proceedings of the Sixty-First Western Poultry Disease Conference.

