# Supplementary material for: First Detection and Identification of FAdV-8b as the Causative Agent of an Outbreak of Inclusion Body Hepatitis in a Commercial Broiler Farm in Greece
Source: Vet Sci. 2022 Mar 25;9(4):160. doi: 10.3390/vetsci9040160 (PMC9027271; doi:10.3390/vetsci9040160)
Supplement: Supplementary file 1 [file vetsci-09-00160-s001.zip › vetsci-1608596-supplementary.pdf]

veterinary sciences

MDPI

Article

First isolation and identification of FAdV-8b as the causative agent of an outbreak of Inclusion Body Hepatitis in a commercial broiler farm in Greece

Vasilios Tsiouris[1], Tilemachos Mantzios[1*], Konstantinos Kiskinis[1], Jean-Luc Guérin[2,3], Guillaume Croville[2,3], Georgia D. Brellou[4], Emmanouela P. Apostolopoulou[4], Evanthia Petridou[5] and Ioanna Georgopoulou[1]

Table S1. Available epidemiological data, for the total of 42 reference strains which were used for the construction of the phylogenetic tree in this study.

	Acc. No.	FAdV-Group	Strain ID	Country	Year (Collection)	PMID
1	EU861033	**A**	PA62/06	Italy	2006	
2	AF339914	**A**	CELO	-	-	19184959
3	EF685377	**A**	05-50052-2324-2H	Canada	2005	--
4	KR259656	**A**	FadV Wroclaw 2015	Poland	2015	-
5	EF685396	**A**	04-42605	Canada	2007	21088178
6	FN557181.1	A	09-1567-9	Germany	2009	-
7	JN181575.1	**A**	K181/10	South Korea	2010	22499868
8	EF685382.1	**A**	05-34275-1837H	Canada	2005	-
9	GU952110.1	**A**	PL/060/08	Poland	2008	21489943
10	AF508952.2	B	340/ EU FAdV 5	-	-	15276983
11	AF508953.1	B	TR22/ EU FAdV 5	-	2002	15276983
12	AF339919.1	B	ATCC VR-830; T8-A	-	2001	19184959
13	FN869988.1	B	09-7473-2	Hungary	2009	20869988
14	EU979371.1	B	340/ FAdV 5		2009	19036935
15	FN869987.1	B	09-7470-2	Hungary	2009	20869988
16	FN869991.1	B	09-6893	Hungary	2009	20869988
17	FN869990.1	B	08-8669	Austria	2008	20869988
18	FN869989.1	B	08-21472	Austria	2008	20869988
19	KX269146.1	C	HeNanMZQ	China	2015	-
20	KT999720.1	C	Shandong	China	2015	-
21	AF339924.1	C	ATCC VR-835; X-11A	-	-	19184959
22	KX179505.1	C	1877570	Pakistan	2015	-
23	AF339917.1	C	ATCC VR-829; J2-A	-	-	19184959
24	AF508950.1	C	506/ EU FAdV 4	-	-	15276983
25	MG765466.1	C	ID-HCI-036	Peru	2016	-
26	GU108581.1	C	-	-	-	-
27	KX247375.1	D	1877566	Pakistan	2015	-
28	AF339916.1	D	ATCC VR-828; IBH-2A	-	-	19184959
29	AF508958.2	D	764/EU FAdV 9	-	-	15276983
30	AF508959.2	D	C2B	-	-	15276983
31	DQ323986.1	D	Stanford	USA	2005	17461263
32	AF339915.1	D	ATCC VR-827; P7-A	-	-	19184959
33	AF339920.1	D	X11	-	-	19184959
34	AF508946.1	D	SR48	-	-	15276983
35	AF508948.2	D	SR49	-	-	15276983

36	JF917237.1	E	UPMPH04217	Malaysia	2004	-
37	EF685497.1	E	04-53357-113	Canada	2004	-
38	KX247373.1	E	2024683	Pakistan	2015	-
39	AF508954.2	E	CR119	-	-	15276983
40	AF508956.2	E	TR59	-	-	15276983
41	MG765463.1	E	ID-HCI-023	Peru	2015	
42	AF339922.1	E	ATCC VR-832; B-3A	-	-	19184959
